# Juvenile Generalized Myasthenia Gravis With AChR and MuSK Antibody Double Positivity: A Case Report With a Review of the Literature

**DOI:** 10.3389/fped.2022.788353

**Published:** 2022-05-11

**Authors:** XiuShan Ge, CuiJie Wei, Hui Dong, YueHua Zhang, XinHua Bao, Ye Wu, DanYu Song, HongJun Hao, Hui Xiong

**Affiliations:** ^1^Department of Pediatrics, Peking University First Hospital, Beijing, China; ^2^Department of Neurology, Peking University First Hospital, Beijing, China

**Keywords:** myasthenia gravis, juvenile, acetylcholine receptor, muscle-specific receptor tyrosine kinase, rituximab

## Abstract

Myasthenia gravis is an autoimmune disease mediated by B cells and is associated with acetylcholine receptor (AChR) and muscle-specific receptor tyrosine kinase (MuSK) antibodies in the postsynaptic membrane at the neuromuscular junction. The presence of both antibodies in the serum of patients with myasthenia gravis has been rarely reported. Case description: A 9-year-old girl was admitted to our hospital with the chief complaints of reduced facial expression for 3 months and unclear speech and choking from drinking water for 2 months. The diagnosis of generalized myasthenia gravis was made based on clinical manifestations, repetitive electrical nerve stimulation, neostigmine tests, specific antibody tests and other auxiliary examinations. We found the rare coexistence of two key antibodies (anti-AChR and anti-MuSK antibodies) in the patient's serum. The patient experienced myasthenic crisis and received respiratory support even though she was taking prednisone therapy. Due to the poor response to treatment with pyridostigmine bromide, glucocorticoids and IVIG, we administered rituximab therapy, and she responded well and achieved clinical remission. This suggests that clinicians should pay more attention to atypical cases and antibody detection. Rituximab should be considered when conventional treatment fails.

## Introduction

Autoimmune myasthenia gravis (MG) is an antibody-mediated chronic disorder in which altered neuromuscular transmission leads to skeletal muscle weakness and fatigability (1). Juvenile MG shares many pathophysiologic and clinical features with adult autoimmune MG but displays distinct demographic patterns, clinical features, and therapeutic challenges and appears to be common in China. It was reported that most antibodies target acetylcholine receptor (AChR, 80%) and less frequently muscle-specific kinase (MuSK, 1–5%) or low-density lipoprotein receptor-related protein 4 (LRP4, 1–33%) (2). The subgroup classification of MG includes clinical feature-based subgroups, such as ocular MG and general MG, or autoantibody-based subgroups, such as AChR-MG, MuSK-MG, LRP4-MG, seronegative MG and thymoma-associated MG. Compared with anti-AChR MG, anti-MuSK MG is much rarer in children, and patients have unique clinical manifestations: the bulbar, facial, neck and respiratory muscles are commonly involved; and they have different reactions to acetylcholinesterase inhibitors (AChEIs), which often need to be combined with immunosuppressive agents.

To date, there have been few reports on the coexistence of dual AChR and MuSK antibodies in MG; this situation is even rarer in children with MG, and no more than 10 cases have been reported globally based on an online database search (3). The clinical characteristics of children with MG with double-positive antibodies are not clear. In this report, we present a rare case of juvenile MG with AChR and MuSK antibodies coexisting at onset before immunotherapy with sufficient follow-up and available clinical details combined with a literature review.

## Case Presentation

A 13-year-old Chinese girl had been admitted to our hospital 3 years and 8 months ago with manifestations of reduced facial expression, unclear speech, and choking cough when drinking water for 3 months. The symptoms had no obvious fluctuations. She did not have significant limb weakness. She was previously diagnosed with epilepsy, and carbamazepine (12 mg/kg) had been used to control seizures well for more than 10 years. Gene testing using next-generation sequencing revealed that the patient carried a heterozygous frameshift variant [NM_145239: c.641dupC (p. Arg217Profs^*^8)] and a heterozygous nonsense variant [c.1011C>T, p.Gly337Gly] of PRRT2 gene, which were inherited from her mother and father, respectively. Normal primary motor milestones were reported.

During the physical examination, the girl showed multiple cranial nerves with innervating muscle weakness, including the 5^th^, 7^th^, 9^th^, 10^th^, and 12^th^ nerves, and limb muscle strength was normal. The bilateral knee tendon reflex was normal. The Babinski sign was negative. In addition, her intelligence was normal. Acute disseminated encephalomyelitis was suspected at first, and she received IVIG at a local hospital and showed no improvement. We performed a neostigmine (0.03 mg/kg) test, which was positive. Then, nerve repetitive electrical stimulation (facial nerve and left median nerve) showed attenuation > 10%, not only in the facial nerve but also in the median nerve, indicating generalized neuromuscular junction involvement. Acetylcholine receptor antibody was positive (value of AchR Ab was 0.987, reference range <0.625), and MuSK antibody was also positive (value of MuSK Ab was 0.697, reference range <0.493), as detected by enzyme-linked immunosorbent assay (ELISA) using ELISA kits (Euroimmun AG, Lubeck, Germany). The levels of antibodies during treatment are shown in Table 1. Mycoplasma pneumoniae antibody titer 1:320. Other etiological tests, thyroid function, cerebrospinal fluid routine and biochemical tests, mediastinal CT and brain MRI were normal.

**Table 1:** Comparison of antibody levels at different treatment stages in this case.

**Antibody types**	**Before the treatment**	**After routine treatment**	**After first round RTX**	**After 2 years of treatment**
AChR Ab(reference range <0.625)	0.987**↑**	0.666**↑**	0.292	0.607
MuSK Ab(reference range <0.493)	0.697**↑**	0.265	0.187	0.150

*AChR Ab, Acetylcholine receptor antibody; MuSK Ab, Musculoskeletal receptor tyrosine kinase antibody, Routine treatment, Full dose of pyridostigmine, IVIG and high-dose intravenous methylprednisolone. RTX, Rituximab. ↑ Means rising level*.

Due to the neostigmine test and repetitive stimulation testing, we gave a localized diagnosis of neuromuscular junction (NMJ). Due to the disease course and autoimmune antibody testing, we diagnosed her with general-type juvenile MG. Therefore, we first administered pyridostigmine bromide (6 mg/kg.d) orally, IVIG and intravenous methylprednisolone (20 mg/kg.d × 3 d, orally 3 days later) during hospitalization. After 3 weeks of initial treatment, the patient's symptoms improved, and the patient was discharged from the hospital. The dosages of pyridostigmine bromide and prednisone were gradually reduced. However, 6 months later, the child's situation was aggravated for respiratory tract infection, and she presented crisis, including difficulty opening her eyes, weakness in breathing and limb weakness due to infection. The muscle strength was reduced to III, and even though the MuSK antibody had decreased to normal, the AChR antibody was still positive. She underwent tracheal intubation and ventilator support at a local hospital. It was proposed that she was not responding to pyridostigmine bromide or corticosteroids, and she was admitted again and treated with rituximab (375 mg/m^2^, once a week, 4 times during the recovery period).

**Figure 1:**
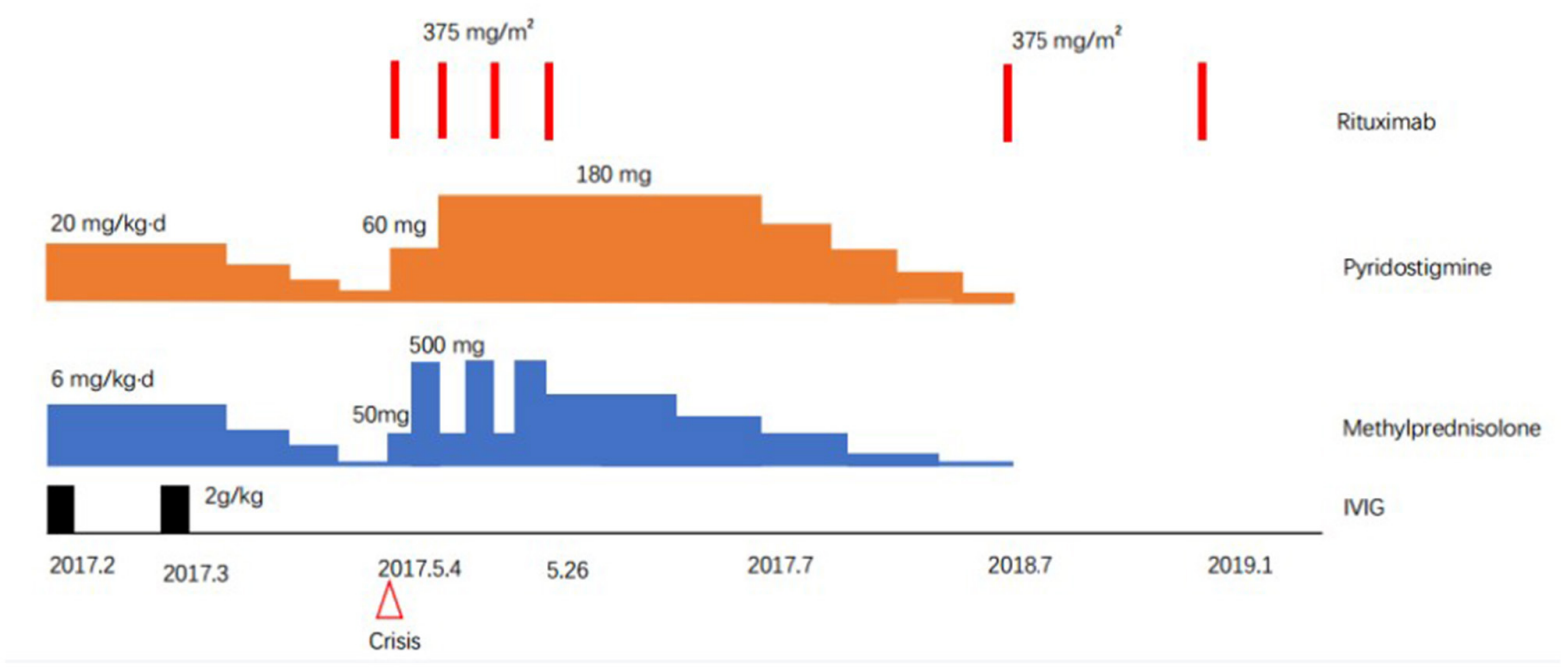
Treatment course of the patient (girl, 13 years old) including Rituximab, methylprednisolone, pyridostigmine, and intravenous immunogloblin (IVIG).

Her facial expression, language, swallowing functions, and muscle strength returned to normal 1 month later. To date, a total of 6 regular rituximab treatments (375 mg/m^2^) have been performed, with a clinical follow-up of ~3 years (during this period, pyridostigmine bromide and methylprednisolone were gradually discontinued). Antibodies related to MG, such as anti-AChR and anti-MuSK antibodies, became negative, and clinical remission was achieved (in Figure 1).

## Review of the Literature

Only 9 cases have been reported to have double-positive antibodies against AChR and MuSK in children worldwide (Table 2) (4–9). Most of these patients were female. The average age of onset was 5.6 years.

**Table 2:** Previous reported pediatric cases of AChR+MuSK-MG.

	**Age of onset (y) /Sex**	**Clinical features**	**MG Crisis**	**AChR-Ab**	**MuSK-Ab**	**RNS**	**TE/ pathology**	**Treatment**	**Evolution**	**References**
1	2/F	Diplopia, bulbar weakness, ptosis, facial muscle weakness, respiratory distress, proximal muscles weakness	+	+	+ after TE	+	+/ UnK	Pyridostigmine, prednisone, PLEX, IVIg	Good improvement	(4)
2	2/F	Respiratory distress, lower extremities muscles weakness, bulbar weakness, neck muscle weakness	+	+	+	+	Thymus not identified	Pyridostigmine prednisone, azathioprine	Good improvement with prednisone	(5)
3	13/F	Ptosis, diplopia, mild extremities muscles weakness After TE, bulbar weakness, neck muscle weakness and respiratory distress	−	+	+ after TE	+	+/follicular hyperplasia	Pirydostygmine (incomplete improvement), prednisone, methylprednisolone and azathioprine.	Clinical improvement	(6)
4	13/F	Diplopia, difficulty swallowing, generalized weakness. After TE, diplopia, facial, bulbar and neck muscle weakness	+	+	+ after TE	+	+/ UnK	Pyridostigmine, prednisolone, IVIG, PLEX, Azathioprine, MMF	Clinical improvement	(7)
5	7/F	Axial weakness and respiratory symptoms, ptosis and diplopia	+	+	+ after TE	−	+/Normal	Pyridostigmine, PLEX, prednisone, MMF	Clinical improvement	(8)
6	4/F	Blepharoptosis, dysphagia, and dyspnea	+	+	+	+	−/ UnK	Pyridostigmine, Corticosteroid	Parital improvement	(9)
7	1/M	Blepharoptosis, ptosis, and dyspnea	+	+	+	−	−/ UnK	Pyridostigmine, Corticosteroid	Good improvement	(9)
8	9/F	facial muscle, bulbar weakness, respiratory muscle, extremities muscles weakness	+	+	+	+	−/ UnK	Pyridostigmine, prednisone, IVIg, rituximab	Good improvement	Our case

*F, Female; M, Male; MG, Myasthenia gravis; AchR, acetylcholine receptor antibody; MuSK, Muscle-specifific kinase autoantibody; TE, Thymectomy; RNS, Repetitive nerve stimulation test; PLEX, Plasma exchange; IVIg, IV immune globulins; MMF, Mycophenolate Mofetil; UnK, unknown*.

## Discussion

This school-age girl had some clinical features that were inconsistent with typical myasthenic gravis, such as symptoms at onset with facial muscle involvement, including reduced facial expression and unclear speech; manifestations of other cranial nerve-innervated muscle involvement, such as weak voice, dysarthria, choking, deglutition and mastication difficulty; respiratory muscle weakness, even including myasthenic crisis after infection; weakness in the limbs that presented as the disease progressed; and symptom aggravation after infection without obvious fluctuations. Her definitive diagnosis was based on electrophysiologic studies and antibody tests.

AChR Ab-positive MG (AChR-MG) and MuSK Ab-positive MG (MuSK-MG) are distinct immunological entities with differences in clinical features, electrophysiological findings, thymus pathology, therapeutic responses and prognosis (10). There are rare reports of patients, especially children, who were found to have elevated titres of both antibodies at onset. Here, we report a girl who had atypical MG manifestations of facial weakness and progressed to myasthenic crisis. She was found to have dual-antibody positivity at onset and responded well to rituximab.

The different types of antibodies produced against the AChR and MuSK antigens in the postsynaptic membrane of MG patients are important biomarkers that guide the diagnosis and treatment of MG. The recent progress of immunological and pathological studies in MG promises to improve myasthenia gravis treatment *via* the development of more precise and personalized therapies. AChR is the most common antigen on the postsynaptic membrane of MG patients, followed by MuSK, which is involved in the aggregation of AChR and the maintenance of the function of neuromuscular junctions (11). The combination of AChR antibodies and antigens is mediated by complement, while MuSK antibodies can directly bind to the antigen to cause disease. Cell-based assays (CBAs) were shown to improve the detection of both antibodies in patients with MG with the highest sensitivity (12). However, ELISA is still used in the clinic and shows high specificity (13). Less than 10 cases have been reported to have double-positive antibodies against AChR and MuSK in children worldwide. Most of them are female. The distribution of myasthenic weakness (facio-pharyngeal muscle involvement with limb sparing) and rapidly evolving clinical deterioration during febrile illness resulting in myasthenic crisis are similar in most cases. The high incidence rate of myasthenia crisis may be features of these double antibody-positive cases. However, the clinical features and diagnostic usefulness of antibodies need to be further studied.

Positive results for both AChR and MuSK antibodies in MG patients at the same time can be seen in two situations: first, with the aggravation of the disease after thymectomy, positive results for both AChR and MuSK antibodies can be seen on the basis of positive AChR antibodies. The pathogenesis of this situation may be antigen drift and immune cross-reaction. In MG after thymectomy, antigen epitope diffusion will cause serum conversion and lead to the occurrence of double-positive antibodies, which has been supported by animal models (14). Second, when antibodies were detected at onset or aggravation, they showed double-positive antibodies, and there was no significant correlation with thymectomy. Such conditions are due to the loss of immune tolerance due to changes in one's own molecular structure or may be due to biochemical changes. The epitope diffusion mechanism causes the development of several antibodies in the same patient. The phenomenon of epitope diffusion suggests that a preliminary autoimmune attack on an additional synaptic epitope can result in damage to the postsynaptic AChR. These epitopes are then processed by antigen-presenting cells and have an impact on the generation of new immunogenicity determinants (15). In this case, the patient had not undergone thymectomy, so they may have experienced the second situation. In addition, the conversion of AChR antibody into MuSK antibody also exists. Potential conditions promoting seroconversion include medication, bacterial or viral infections, thymectomy and unknown factors. A complete serological examination of the suspected patient would shed light on the problem.

The response to treatment is different, and most patients are refractory to standard first-line therapy. Compared with AChR-MG, MuSK-MG has a poor response to cholinesterase inhibitors, and the conventional dose of pyridostigmine can cause significant side effects. MG has different responses to glucocorticoids (16). In only one study, rituximab treatment in 2 children showed significant efficacy, which should be given more attention.

Rituximab (RTX), a monoclonal antibody that leads to the rapid depletion of B cells and their precursors, has emerged as a potentially efficacious off-label treatment option for patients with MG, especially refractory MG. RTX is an appealing option due to its mechanism of action targeting CD20-positive B cells, which are involved in the disease process of MG. Most studies emphasize that rituximab has obvious advantages in MG treatment, among which the effect on MuSK-MG is more significant than that on AChR-MG due to its pharmacological mechanism (17). Long-term follow-up is necessary, and RCT studies are still needed to confirm the therapeutic effect of RTX in different antibody subtypes of MG in the future (18). Through the treatment of this patient, we found that the antibody titer was not always proportional to disease severity, though glucocorticoids may play a role in returning the MuSK antibody level to normal. Rituximab is faster and more stable than cholinesterase inhibitors and glucocorticoids in alleviating symptoms, and it has obvious advantages in the discontinuation of glucocorticoids and immunosuppressive agents. In addition, rituximab has a better effect on the normalization and maintenance of AChR and MuSK antibody levels. Of course, in the long term, we still need to pay attention to the duration, withdrawal time and safety of rituximab.

In conclusion, we report a case of GMG in a child with a special antibody composition. The diagnosis and treatment were comprehensive, and the outcome was satisfactory. In the detection of double-positive AChR and MuSK Abs, physicians often feel unsure about the optimal treatment strategy and long-term prognosis. Detailed clinical records and long-term observations of these rare cases are needed for best management in clinical practice. The application of other immunosuppressants should be carefully considered for pediatric patients with unsatisfactory responses to first-line treatment, especially when rituximab has a significant therapeutic effect. The long-term prognostic significance of this immunological coexistence is still unknown and difficult to establish because of its rarity. Meanwhile, the targeted identification of MG antibody subtypes in children and summary of their clinical characteristics may be of great significance for improving the understanding of MG.

## Data Availability Statement

The original contributions presented in the study are included in the article/supplementary material, further inquiries can be directed to the corresponding author.

## Ethics Statement

The research protocol (2015[916]) was reviewed and approved by the Ethics Committee of the Peking University First Hospital (Beijing, China). Written informed consent, which also included consent for the publication of medical data, was obtained from the patient and her parents.

## Author Contributions

XG and HX wrote the first draft of the manuscript and were in charge of the patient's follow-up. CW, HD, YZ, XB, and YW provided neurologic management. DS was in charge of the patient's follow-up. HH provided scientific contributions and critically revised the manuscript. All authors have read and approved the final version of the manuscript.

## Funding

This study is supported by grants from the National Key Research and Development Program of China (No. 2016YFC0901505).

## Conflict of Interest

The authors declare that the research was conducted in the absence of any commercial or financial relationships that could be construed as a potential conflict of interest.

## Publisher's Note

All claims expressed in this article are solely those of the authors and do not necessarily represent those of their affiliated organizations, or those of the publisher, the editors and the reviewers. Any product that may be evaluated in this article, or claim that may be made by its manufacturer, is not guaranteed or endorsed by the publisher.
